# The Universities in Research and in Practice

**Published:** 1920-07-10

**Authors:** 


### THE UNIVERSITIES IN RESEARCH AND IN PRACTICE.

In a really excellent Presidential Address at the inauguration of the scientific sections of the recent B.M.A. meeting at Cambridge, Sir Clifford Allbutt gave an arresting picture of the vital part which the universities have played in the improvement of medical practice and the development of research. It is almost impossible to convey in a few words the many lessons which his intimate knowledge of the medical profession allowed him to provide, and at best we can but select a sentence here and there which may bring them home to those who have not time or opportunity to study the full text of his speech.

To every student the university has stood for something larger than the field of a particular calling, for a more comprehensive design. Conversation in universities has kept the mind open and flexible, and has taught that no craft is limited to the material with which it deals. We must be trained not only how to learn particulars, but also how to handle them. Tacitus told us that no man should apply his mind to some particular profession till he had extended and strengthened it by general culture, even as earth is not sown until it has been turned and turned again several times. We now try to build up, first, character, the quality which has governed the world; secondly, to train in clear thinking and to learn not to go beyond the evidence; thirdly, to impart particular knowledge and experience. But we should hope to nourish yet another and still more precious quality than these. Nowadays the memory is exercised, the intellect is more or less called upon, but the centre of creative light and action—the imagination—is too often left out in the cold. The old university builders built for the imagination.

Again, turning to research in particular subjects, the President deplored isolation in scientific work when applied, for example, to comparative pathology. Year after year enormous loss is caused by the depredations and the diseases of animals and plants, and many of the methods of dealing with these trials are barbarous, if at present imperative. Yet, as Sir Clifford Allbutt says, no one stirs save to gyrate each in his own little circle. There has been no integration, no organisation of research, no cross-light from school to school, no mutual enlightenment among investigators, no big outlook. Diseases are not "entities" nor even recurrent phases of independent events, but partial aspects of a universal series. The young graduates we have are many of them of great capacity; but every day we are losing them because they are not taken up at once into scientific teams; so they slacken, or drift into some other means of livelihood, and things muddle on as before.

If we would consider general practice and trace the necessity for united and understanding co-operation on the part of practitioners concerned, and see therein indirectly reflected the university spirit, we should at the same time note Sir Clifford Allbutt's favourable comment on an American development. "In the United States medical men are banding together in districts for such development of their private practices. Five or six of them combine to rent a house, with consulting rooms for each, and a common apartment for minor surgery, etc. Each member of the alliance—they are not exactly partners—is expected, in addition to general practice, to take up special work in some department supplementary to the others; so that a fair variety of special skill may be available in the house; skill if not of high expert value, which must be sought elsewhere, yet quite sufficient for ordinary diagnosis and treatment. . . Patients like the system; they see that it is more thorough." No doubt such ends may be attained by different methods, but the urgent need is the generally accessible laboratory and staff, which is to be a little academy and a place of reunion—a centre with the university ideals. Every day it is becoming plainer to the public that the treatment of the sick must be fortified with, if not actually based upon, the ancillary sciences. No university can have a nobler faculty than medicine, and in no calling is the university spirit and training from first to last more necessary than the profession of the medical man.

